# Biodistribution and blood clearance of plasmid DNA administered in arginine peptide complexes

**DOI:** 10.1186/1479-0556-9-13

**Published:** 2011-08-17

**Authors:** Jung Gyu Woo, Na Young Kim, Jai Myung Yang, Sungho Shin

**Affiliations:** 1 Department of Life Science, Sogang University, Shinsu-Dong, Mapo, 121-742, Seoul, Republic of Korea

**Keywords:** Arginine peptide, Biodistribution, Gene therapy, Peptide vector, Systemic gene delivery

## Abstract

**Background:**

Peptide/DNA complexes have great potential as non-viral methods for gene delivery. Despite promising results for peptide-mediated gene delivery technology, an effective systemic peptide-based gene delivery system has not yet been developed.

**Methods:**

This study used pCMV-Luc as a model gene to investigate the biodistribution and the *in vivo *efficacy of arginine peptide-mediated gene delivery by polymerase chain reaction (PCR).

**Results:**

Plasmid DNA was detected in all organs tested 1 h after intraperitoneal administration of arginine/DNA complexes, indicating that the arginine/DNA complexes disseminated widely through the body. The plasmid was primarily detected in the spleen, kidney, and diaphragm 24 h post administration. The mRNA expression of plasmid DNA was noted in the spleen, kidney, and diaphragm for up to 2 weeks, and in the other major organs, for at least 1 week. Blood clearance studies showed that injected DNA was found in the blood as long as 6 h after injection.

**Conclusions:**

Taken together, our results demonstrated that arginine/DNA complexes are stable in blood and are effective for *in vivo *gene delivery. These findings suggest that intraperitoneal administration of arginine/DNA complexes is a promising tool in gene therapy.

## Background

Cell-penetrating peptides (CPPs) have been widely shown to transfer macromolecules into living cells [1,2]. Several of these peptides have been identified, such as Tat [3], Antp [4], and VP22 [5]. Carrier peptides, which are fused to their cargo molecules, provide a method for delivering intracellularly acting proteins or nucleic acids to cells *in vitro *[6,7], *ex vivo *[8], and *in vivo *[9,10]. For example, it was recently reported that CPPs are highly efficient in facilitating the cellular uptake of small interfering RNA (siRNA) [11,12]. Most CPPs contain at least 1 basic amino acid residue such as arginine or lysine, suggesting that basic amino acids are critical motifs for the efficient delivery of exogenous biomolecules into cells [13,14].

The authors have focused on the development of an arginine peptide-mediated gene delivery system after previously demonstrating that a short arginine peptide (R15) is able to condense plasmid DNA into small complexes. The highest transfection activity in 293T, HeLa, Jurkat, and COS-7 cells was obtained for arginine/DNA complexes with an N/P ratio of 3:1 [15]. The size of the arginine/DNA complex was shown to be the primary limitation for transfection efficiency *in vitro *[16]. Confocal laser fluorescence microscopy data showed that arginine peptides facilitated the movement of DNA from the cytoplasm, causing DNA to accumulate in the nucleus [17].

The success of gene therapy depends on the development of a vector that achieves efficient, cell-specific, and prolonged transgene expression after its application [18]. Although viral vectors have the highest transfection efficiency among the many possible gene carriers, safety concerns have led to reconsideration of their use in human gene therapy. Non-viral vectors such as cationic peptides are considered safer and easier to prepare than viral vectors, and are, therefore, more attractive vectors for clinical application of gene therapy [19]. Despite their usefulness, there has been little systemic *in vivo *study of peptide vectors. More importantly, studies on the pharmacological profile of intraperitoneally administered arginine/DNA complexes are completely lacking. Determining critical pharmacological parameters such as plasmid biodistribution, blood clearance half-life, *in vivo *persistence, and gene expression is very important in the design of new delivery strategies.

Therefore, the objective of this study was to assess the *in vivo *fate of arginine/DNA complexes after their intraperitoneal administration in mice using luciferase as a reporter gene. Organ distribution in terms of plasmid localization, DNA expression, and circulation kinetics were assessed. Polymerase chain reaction (PCR) was employed to assess plasmid DNA and expression of DNA in the different organs.

## Methods

### Plasmid DNA

Plasmid DNA containing firefly luciferase under the control of a CMV-promoter (pCMV-Luc) was provided by Promega (Madison, WI, USA). The plasmid DNA was amplified in *Escherichia coli *TOP10-competent cells and purified with an AxyPrep™Plasmid Maxiprep Kit (Union City, CA, USA), according to the manufacturer's instructions. The quality of plasmid DNA preparations was determined using NanoDrop ND-1000 (Wilmington, DE, USA). Typical optical density (O.D.) at 260/280 nm values were approximately 1.9. DNA was stored at -20°C until use.

### Formation of arginine/DNA complexes

Arginine/DNA complexes were generated at an N/P ratio of 3:1, as described previously [15]. Plasmid DNA (100 μg) was added to a 5% glucose solution and 6.1 μL of 10 mM arginine peptide (R15; Peptron, Daejeon, Korea) was added to the final 5% glucose solution and adjusted to a final volume of 500 μL. To form the arginine/DNA complexes, the solution was pipetted and vigorously mixed by vortexing. The complex solution was incubated for 15 min at room temperature (25°C) and intraperitoneally administered to the mice.

### *In vivo *gene delivery

All animal work was conducted according to the guidelines established by the Institutional Animal Care and Use Committee of the Sogang University. Female Balb/c mice (Samtako, Osan, Korea) weighing 19-20 g (5-week-old) were used for *in vivo *gene delivery. Five hundred microliters of the arginine/DNA complex (N/P ratio of 3.0; 100 μg pCMV-Luc) in 5% glucose solution was administered by intraperitoneal injection with a 27-gauge syringe needle.

### Biodistribution studies

For biodistribution experiments, blood was collected from the vena cava of Balb/c mice intraperitoneally injected with the arginine/DNA complex solution under ether anesthesia at the indicated time points, and the mice were subsequently killed by cervical dislocation. The organs (liver, lung, heart, spleen, brain, diaphragm, and kidney) were removed. Samples were thoroughly washed with phosphate-buffered saline (PBS) to minimize the influence of plasmid in the blood, blotted dry, and weighed. Blood samples were treated with heparin (Sigma, St. Louis, MO, USA) to prevent aggregation.

### Isolation of DNA and RNA

At various time points following intraperitoneal administration of arginine/DNA complexes, samples of several tissue types were obtained, including the liver, heart, spleen, brain, diaphragm, kidney, and blood. Subsequently, samples were homogenized using a BioMasher (Nippi, Tokyo, Japan) or a glass homogenizer. The DNA was purified using the DNeasy Blood and Tissue Kit (Qiagen, Valencia, CA, USA) protocol. Total RNA was extracted from each sample using the RNeasy Mini Kit (Qiagen).

### PCR detection of plasmid DNA

PCR was used to visualize reporter gene biodistribution to each organ. The primers used in the reactions were as follows: luciferase forward primer 5'-tgcactgatcatgaactc-3' and reverse primer 5'-ggacataatcataggacc-3'. The reactions were set up using 50 ng of total DNA and 2 × Premix Taq (Takara, Seoul, Korea). The PCR process was controlled by a MasterCycler (Eppendorf, Hamburg, Germany) as follows: pre-incubation at 94°C for 5 min, 40 cycles of denaturation at 94°C for 30 s, annealing at 50°C for 30 s, extension at 72°C for 40 s, and post-amplification at 72°C for 7 min. Nested PCR was used to examine blood clearance and the duration of mRNA expression. Reactions were constructed as an additional nested PCR after the first PCR. The nested PCR reaction was constructed as follows: luciferase nested forward 5'-cgctgctggtgccaaccc-3' and luciferase nested reverse 5'-tttaccgaccgcgcccgg-3' primers, template, 3 μL of the first PCR product, and 2 × Premix Taq. The second PCR thermal cycle was the same as the first, except that the annealing and extension temperatures and times were 62°C for 30 s and 72°C for 20 s, respectively. The PCR products were visualized using 1.2% agarose gel electrophoresis.

### Reverse transcription PCR (RT-PCR) assay

To determine the mRNA expression of the administered plasmid DNA in various organs, mRNA levels were measured using RT-PCR. To prepare the cDNA templates, 2 μg of total RNA from each organ were used as a template for reverse transcription using AccuPower RT Premix (Bioneer, Daejeon, Korea) with Oligo dT as a primer for reverse-transcriptase. The cDNA was synthesized at 70°C for 10 min, at 42°C for 1 h, and at 94°C for 5 min.

### Relative quantification of reporter gene mRNA

The real-time PCR reaction for relative quantification of luciferase mRNA was performed in 20-μL reaction volume containing 0.5 μL of luciferase nested forward and reverse primers, 2 μL template cDNA, 0.4 μL ROX reference dye, and 10 μL of 2 × SYBR Premix Ex Taq (Takara, Seoul, Korea). The thermal cycler protocol was set as follows: pre-incubation at 95°C for 10 s, amplification at 40 cycles at 95°C for 5 s, and 60°C for 40 s. For the mouse glyceraldehyde-3-phosphate dehydrogenase (GAPDH) cDNA measurements, each sample was prepared following the manufacturer's instructions with a GAPDH primer set (Qiagen). Relative quantification was expressed as the SYBR fluorescence ratio as luciferase fluorescence/GAPDH fluorescence.

## Results

### Biodistribution of intraperitoneally administered plasmid DNA

**Figure 1:**
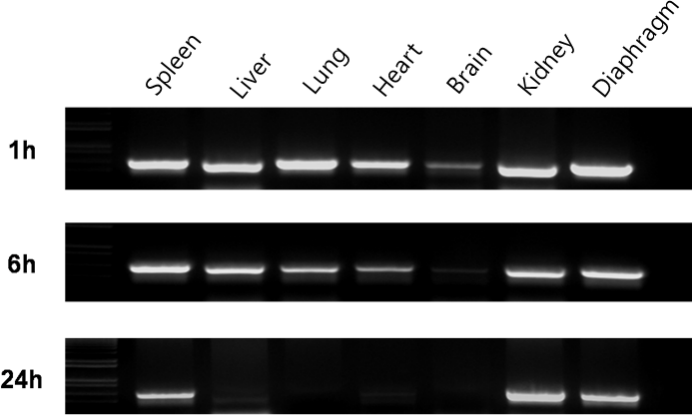
**Organ distribution of plasmid DNA and the time course of its clearance after delivery in arginine/DNA complexes**. Plasmid DNA (100 μg) complexed with arginine peptide at an N/P ratio of 3:1 was intraperitoneally administered to mice. The DNA was analyzed by PCR for the luciferase transgene from various organs by using the specific primers described in the Materials and Methods section. The PCR products were separated on a 1.2% agarose gel.

The biodistribution of the plasmid DNA was studied after intraperitoneal administration in mice using PCR analysis. pCMV-Luc was chosen as a target plasmid. Mice were injected with arginine/DNA complexes prepared with 100 μg plasmid DNA at an N/P ratio of 3:1 and were sacrificed at various time points. Plasmids were found in the spleen, liver, heart, lung, kidney, brain, and diaphragm 1 h after administration (Figure 1). Notably, plasmid distribution to the brain was comparable to that to the other organs. Plasmid DNA was still present in all organ samples 6 h post-dose, but the level of plasmids in the brain was significantly lower than in other organs at this time point. Plasmid DNA was detected only in the spleen, kidney, and diaphragm 24 h after inoculation. These results show that the arginine/DNA complexes had diffused throughout the peritoneal cavity, and that the plasmid DNA was delivered to various organs.

### Quantification of mRNA expression in organs

**Figure 2:**
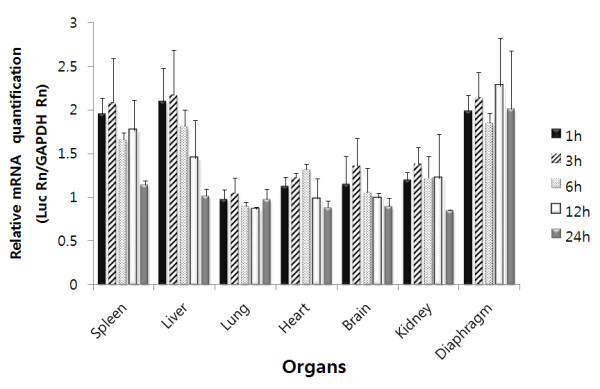
**mRNA expression levels of the target gene in various organs**. mRNA levels were evaluated using real time RT-PCR. Plasmid DNA (100 μg) complexed with arginine peptide at an N/P ratio of 3:1 was intraperitoneally administered to mice. Mice were sacrificed at the indicated time points, and total RNA was extracted from the organs. After preparation of cDNA, PCR amplification of luciferase and GAPDH genes was performed using the specific primers described in the Materials and Methods section. Results are expressed as means ± S.D. for at least 3 different experiments.

To determine whether the plasmid DNA detected in various organs remained sufficiently intact for *in vivo *transcription, the mRNA expression levels of luciferase DNA in the organs were tested. Transgene expression was evaluated using the real time RT-PCR assay. The murine housekeeping gene GAPDH was used as an internal control for the quantitative analysis. mRNA was detected in all of the organs examined as early as 1 h after administration, with high levels of mRNA found in the spleen, liver, and diaphragm, whereas the heart, lung, kidney, and brain showed lower levels of gene expression (Figure 2). Expression levels peaked in the organs 3 h after administration of plasmid DNA. The diaphragm showed the highest level of mRNA expression and retained high levels of mRNA expression until 24 h after administration. However, unlike the diaphragm, the levels of mRNA expression in the other organs decreased rapidly 12 h after administration. These results indicate that the plasmid DNA delivered by peptides to various organs remains sufficiently intact for transcription.

### pCMV-Luc plasmid DNA dose response

**Figure 3:**
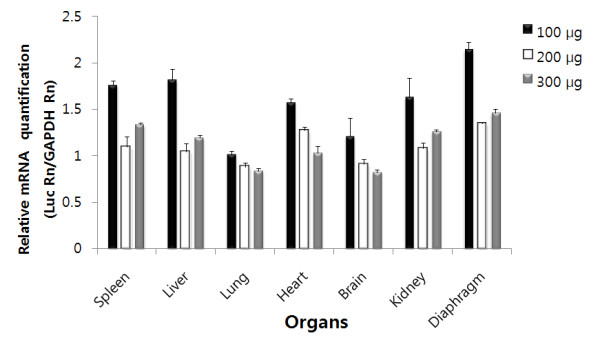
**Effects of DNA dose on plasmid DNA expression after delivery in arginine/DNA complexes**. Various amounts of plasmid DNA complexed with arginine peptide at an N/P ratio of 3:1 were intraperitoneally administered to mice, and mRNA levels were evaluated using real time RT-PCR. Total RNA was extracted from the organs. After preparation of cDNA, PCR amplification of luciferase and GAPDH genes was performed using the specific primers described in the Materials and Methods section. Results are expressed as means ± S.D. for at least 3 different experiments.

DNA dose effect on the level of mRNA expression was also assessed using real time RT-PCR assay. Figure 3 illustrates data obtained when increasing amounts were injected intraperitoneally into mice and the mRNA expression was determined 3 h later. In this experiment, 100, 200, and 300 μg of pCMV-Luc plasmid were complexed with arginine peptide, so that the N/P ratio remained at 3:1. Interestingly, the expression level of target mRNA was not increased in a plasmid DNA dose-dependent manner. A significant level of mRNA expression was detected in all organs, including the spleen, liver, lung, heart, brain, kidney, and diaphragm, when 100 μg of pCMV-Luc plasmid DNA was injected into mice. However, further increasing the plasmid DNA dose to 300 μg did not result in a significantly increased mRNA expression level in the organs. Thus, the observed mRNA expression level appears to saturate at a dose of 100 μg DNA/mouse.

### Duration of plasmid DNA expression

**Figure 4:**
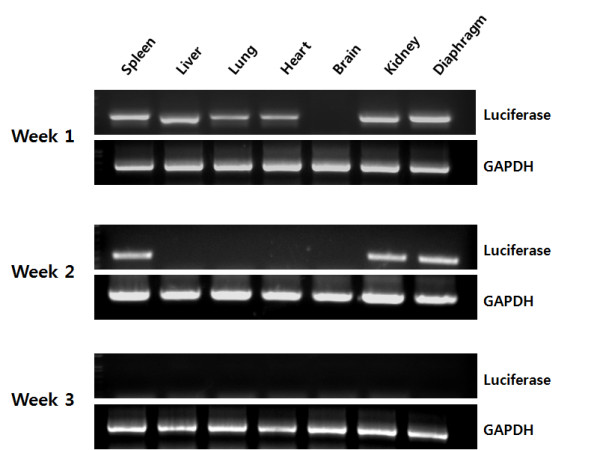
**Duration of plasmid DNA expression**. Plasmid DNA (100 μg) complexed with arginine peptide at an N/P ratio of 3:1 was intraperitoneally administered to mice, and total RNA was extracted from the organs at the indicated time points. The RNA extracts were transformed to cDNA using RT-PCR to serve as templates for nested PCR analysis. PCR amplification of luciferase and GAPDH genes was performed using the specific primers described in the Materials and Methods section. The nested PCR products were separated on a 1.2% agarose gel.

Given the organ distribution and the optimum injection volume results, we next examined the duration of plasmid DNA expression in various organs by nested PCR analysis (Figure 4). Prolonged DNA expression was observed in the spleen, kidney, and diaphragm. All organs tested, except the brain, retained the expression of the administered genes with a high level of mRNA expression of luciferase relative to GAPDH in each organ 7 days after administration. The spleen, kidney, and diaphragm showed high levels of mRNA expression, whereas the other organs did not show detectable levels of mRNA expression 14 days after plasmid DNA application. mRNA expression disappeared substantially in all the tested organs 21 days after administration.

### Blood clearance of plasmid DNA

**Figure 5:**
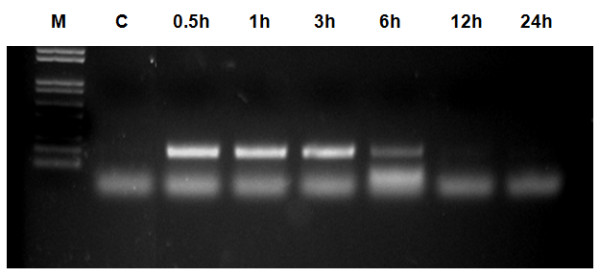
**Blood clearance of plasmid DNA after delivery in arginine/DNA complexes**. Plasmid DNA (100 μg) complexed with arginine peptide at an N/P ratio of 3:1 was intraperitoneally administered to mice. DNA was extracted from blood at the indicated time points and used for nested PCR products. The nested PCR products were separated on a 1.2% agarose gel.

To better understand the pharmacokinetic character of plasmid DNA, its blood clearance profile was studied following intraperitoneal administration. The presence of plasmid DNA was determined at select times by using nested PCR analysis. A PCR band of plasmid DNA was observed in blood samples, which gradually decreased at progressively later time points. Plasmid DNA was detected up to 6 h post administration, whereas lower levels of plasmid DNA were detected in the 12 h blood sample and plasmid DNA was not detected after 12 h (Figure 5). These results indicate that plasmid DNA is stable for at least 6 h in the blood and can circulate in the bloodstream, thereby increasing the opportunity for delivery to target organs.

## Discussion

CPPs have shown efficient *in vitro *transfection efficiency without significant cellular toxicity [1]. Over the past decade, peptide vectors have been shown to be an effective way of delivering DNA into cells, and unlike viral vectors, peptides do not present safety concerns such as immunogenicity and insertional mutagenesis. Peptide vectors are able to compact and protect DNA, enter cells via endocytosis, and deliver DNA cargo to the nucleus [2,14]. Efficient cell-specific delivery of peptide/DNA complexes is a major advantage of peptide vectors. Several small peptides have been described, most notably the tripeptide motif RGD, which targets integrin receptors specially. RGD-containing peptides associated with polylysine significantly improve the delivery of DNA into specific cell lines [20]. Another targeting approach is to use targeting moieties, such as the epidermal growth factor peptide which targets mainly cancer cells, covalently linked to one of the component of the peptide/DNA complex [21]. Although peptide vectors are under intensive investigation as promising vectors for gene therapy, relatively little information is available regarding the *in vivo *pharmacological profiles of administered peptide vectors. In this paper, the performance of a short arginine peptide (R15) vector as a gene carrier was evaluated *in vivo*.

The biodistribution of DNA complexes with arginine peptide after intraperitoneal administration was initially investigated using PCR analysis, indicating that intraperitoneally applied arginine/DNA complexes were absorbed into the systemic circulation and distributed to the major organs of mice. Plasmid DNA was found in all analyzed organs, including the spleen, liver, heart, lung, kidney, brain, and diaphragm. Similar observations have been previously reported by other groups after intraperitoneal injection of polyplex [22] or lipoplex in mice [23,24]. For example, Louis et al. reported that large amounts of plasmid DNA were detected in the kidney, spleen, and diaphragm after intraperitoneal injection of DNA with polyethylenimine [25]. It is notable that low but significant quantities of plasmid DNA were localized in the brain. Recently, it was reported that arginine peptide efficiently facilitates rabies virus glycoprotein (RVG)-mediated brain cell uptake of siRNA [11], and that high brain uptake values were observed for penetratin and Tat [26]. These results suggest that arginine-associated delivery will be useful for the brain-directed transport of therapeutic molecules. Plasmid DNA clearance varied in different organs and the rapid disappearance of DNA from the liver, heart, brain, and lungs suggests that plasmid DNA is locally degraded by nucleases.

The mRNA expression pattern was in good agreement with the plasmid DNA localization data. Significant mRNA expression of the luciferase gene in the plasmid DNA was observed in all of the tested organs (Figure 2). mRNA was detected as early as 1 h after DNA injection, suggesting that the intraperitoneally administered plasmid DNA complexed with arginine peptide was delivered to various organs in a sufficiently intact form for transcription. Similar rapid gene expression was reported in a previous study, in which luciferase activity was detected as early as 3 h after plasmid DNA infusion into mice [27]. In agreement with the pattern of plasmid clearance revealed by PCR analysis, the mRNA expression level was highest in the spleen and diaphragm, in which the longest presence of plasmid DNA was observed. To determine the effect of plasmid dose on mRNA expression, the plasmid DNA dose was increased up to 300 μg. Interestingly, the mRNA expression levels of plasmid DNA did not increase with the increased amounts of plasmid DNA (Figure 3), suggesting that a saturation phenomenon occurred under these experimental conditions. Previous studies have demonstrated that the gene expression level does not correspond with the amount of administered cationic liposome/DNA complexes [28,29].

Prolonged expression of plasmid DNA was observed in arginine/DNA complex-treated mice (Figure 4), which is comparable to the previous observations in naked DNA-treated mice. However, the organs of naked DNA-treated mice did not express mRNA from the topically or intravenously administered genes 3 to 5 days after dosing [30]. In contrast, the results presented herein show that some organs retained high levels of mRNA expression for more than 14 days after application. Prolonged blood circulation of plasmid DNA was also observed in arginine/DNA complex-treated mice (Figure 5), and the blood circulation time in the present study was 6 h. To put this rate in context with other non-viral vectors, polylysine/DNA complexes are cleared from circulation within 5 to 30 min [31,32]. Cationic liposome/DNA complexes are cleared more rapidly, with only 10% of the injected complexes remaining detectable in the blood as little as 1 min after injection [33]. Taken together, these results provide evidence that arginine/DNA complexes are stable for a relatively prolonged time under *in vivo *conditions, which is one of the critical requirements for an efficient gene delivery vector. Furthermore, preferential plasmid distribution was observed in the diaphragm, which presents a peritoneal surface. Tumors in the peritoneal cavity are difficult to detect and cancer often persists despite surgery and other treatments [34]. In case of ovarian cancer, overall 5-year survival rate is very low, mainly as a consequence of late tumor detection (after peritoneal dissemination) and chemoresistance following chemotherapy. Therefore, the efficient peritoneal cavity-preferential gene delivery and prolongation of complex stability under *in vivo *conditions suggest that the intraperitoneal injection of arginine peptide/DNA complexes will play an important role in future gene therapies for peritoneal malignancies.

## Conclusions

In summary, the present findings demonstrate that arginine/DNA complexes are very stable when administered intraperitoneally, and are effective agents for *in vivo *gene delivery. Although optimization studies of these strategies need to be continued, the information presented in this paper will be valuable for the development of peptide-based vectors to enhance the potential of gene therapy. Further studies will be focused on understanding the factors affecting the biodistribution and examining the possibility of targeting specific organs and cell types.

## Competing interests

The authors declare that they have no competing interests.

## Authors' contributions

All authors have read and approved the final manuscript. JGW has performed the *in vitro *and *in vivo *experiments. NYK has helped with the experiments and data presentation. JMY has reviewed the manuscript and data interpretation. SS has designed the experiments, interpreted the results and drafted the manuscript.
